# Endoclips as novel fiducial markers in trimodality bladder-preserving therapy of muscle-invasive bladder carcinoma: feasibility and patient outcomes

**DOI:** 10.1590/S1677-5538.IBJU.2019.0713

**Published:** 2020-11-18

**Authors:** Muhammad Shahbaz, Asif Ammar, Yuliang Wang, Zeeshan Farhaj, Liang Qiao, Jun Niu

**Affiliations:** 1 Shandong University Qilu Hospital Department of General Surgery Jinan China Department of General Surgery, Qilu Hospital, Shandong University, Jinan, 250012, China; 2 Shandong University The Institute of Laparoscopic-Endoscopic Minimally Invasive Surgery Shandong China The Institute of Laparoscopic-Endoscopic Minimally Invasive Surgery of Shandong University, Shandong, China; 3 Weifang People´s Hospital Department of Urology ShandongWeifang China Department of Urology, Weifang People´s Hospital, Shandong, Weifang 261041, China; 4 Combined Military Hospital Department of Urology Kharian Pakistan Department of Urology, Combined Military Hospital, Kharian, Pakistan; 5 Weifang Medical University ShandongWeifang China Weifang Medical University, Shandong, Weifang 261041, China; 6 Shandong University Shandong Provincial Hospital Department of Cardiovascular Surgery Jinan China Department of Cardiovascular Surgery, Shandong Provincial Hospital Affiliated to Shandong University, No. 324 Jingwu Road, Jinan, China

**Keywords:** Urinary Bladder Neoplasms, Carcinoma, Treatment Outcome

## Abstract

**Hypothesis:**

Endoclip can be used as fiducial marker in urology.

**Objective:**

To assess the feasibility, cost effectiveness and reliability of endoclips as novel fiducial markers in precision radiotherapy, as part of a trimodality bladder-preserving treatment (TBPT) of muscle-invasive bladder carcinoma.

**Materials and Methods:**

This retrospective study was performed at Weifang People's Hospital (Weifang, China) from January 2015 to June 2018. A total of 15 patients underwent TBPT. Endoclips were applied to healthy edges of the resected bladder wall as novel fiducial markers. Radio-sensitizing chemotherapy and routine precision radiotherapy were given. The number and position of the endoclips during radiotherapy sessions were monitored. Complications and tumor recurrence were analyzed.

**Results:**

The mean age (±standard deviation) of the patients was 67±10 years (range 46-79). There were 3 females and 12 males. Forty-nine endoclips were applied in all patients (3.3±0.8). The tumor was completely visibly resected in all patients. The number of endoclips remained the same through the planned last radiotherapy session (3.3±0.8), i.e., none were lost. All endoclips were removed after the last radiotherapy session. The average number of follow-up months was 38.9±13.2 (range 11-52). There were no procedure-related complications at discharge or follow-up. At one-year, overall recurrence-free survival was 93.3%. Two patients had recurrences at 18 months and 10 months after TBPT, respectively, and salvage radical cystectomy was performed with no further recurrences. Another patient died due to metastasis 9 months after the completion of therapy.

**Conclusions:**

Endoclips are reliable, safe and cost-effective as novel fiducial markers in precision-radiotherapy post-TBPT.

## INTRODUCTION

Bladder cancer is the 9^th^ most common cancer worldwide, with an annual incidence of 430.000 cases (1). It is ranked 13^th^ in terms of cancer-related mortality (1) and affects up to six times as many men as women (2, 3). It is largely a disease of industrialized nations, and the age-standardized incidence is tjree times higher in developed countries compared to developing countries (4). Muscle-invasive bladder carcinoma accounts for 25% of all bladder cancers (5).

The most common bladder-preserving techniques are transurethral resection with adjuvant radiotherapy, chemotherapy and definitive chemoradiation (6, 7). However, a challenge with radiotherapy is that it may endanger healthy tissue. In bladder cancer, radiotherapy is complicated by inter- and intra-fraction target motion, which can vary up to 3cm and is dependent on the location of the tumor in the bladder (8, 9). Complete bladder treatment and large planning target volume margins, ranging from 15-20mm, are used as a precaution for the target motion as the standard of care (9).

Target visualization is critical in guided radiotherapy and is more challenging in partial bladder radiotherapy (10). Fiducial markers such as gold seeds and Lipiodol are used to improve visualization of the target area. However, both these markers may be lost or may move with time. For example, for Lipiodol, this ranged from 5-24% of markers by the final radiotherapy session (11), and for the gold seeds this ranged up to 25% (12). Furthermore, one study reported that gold seeds are difficult to implant in the dome of the bladder (12), and another study found that Lipiodol was difficult to inject close to the bladder neck (13).

There are no reports on the use of endoclips as fiducial markers in precision radiotherapy of muscle-invasive bladder carcinoma. In this retrospective study, we aimed to assess the feasibility and reliability of endoclips as novel fiducial markers for aid in precision radiotherapy after resection of muscle-invasive bladder carcinoma.

## MATERIALS AND METHODS

This retrospective study was performed at Weifang People's Hospital, Weifang, China. Between January 2015 and June 2018, a total of 15 patients underwent bladder-preserving trimodality transurethral resection of muscle-invasive bladder tumor. All 15 patients were diagnosed with T_2_N_0_M_0_ stage muscle-invasive bladder carcinoma after a comprehensive tumor study that included computed tomography, magnetic resonance imaging, and electronic cystoscopy. The study was approved by the ethics committee on scientific research of our hospital (Approval No. 2014-006). Informed written consent was obtained from all patients or their guardians. Patient characteristics are presented in Table 1. Currently, there are two preferred approaches for T_2_N_0_M_0_ stage muscle-invasive bladder carcinoma: the common choice of radical cystectomy or bladder-preserving trimodality treatment. In this study, we used the bladder-preserving trimodality approach.

**Table 1:** Patient characteristics.

Age [years] (range)		67±10 (46-79)
Sex (F/M)		3/12
Carcinoma in situ		15/15
Clinical T_2_N_0_M_0_ Stage (%)		100%
**Smoker n (%)**		
	Yes	5 (33%)
	No	10 (67%)
Operation time [minutes]		66.6±40.9 (22-152)

### Surgical procedure and chemoradiotherapy

**Figure 1:**
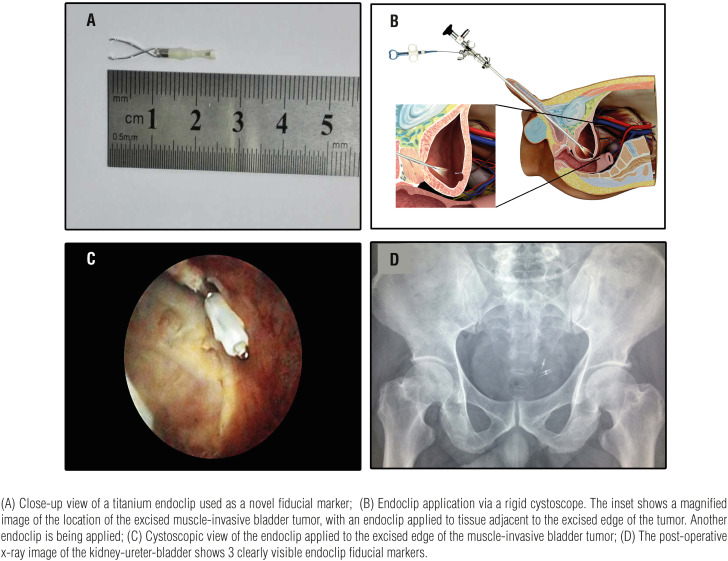
Endoclip Application and follow-up. (A) Close-up view of a titanium endoclip used as a novel fiducial marker; (B) Endoclip application via a rigid cystoscope. The inset shows a magnified image of the location of the excised muscle-invasive bladder tumor, with an endoclip applied to tissue adjacent to the excised edge of the tumor. Another endoclip is being applied; (C) Cystoscopic view of the endoclip applied to the excised edge of the muscle-invasive bladder tumor; (D) The post-operative x-ray image of the kidney-ureter-bladder shows 3 clearly visible endoclip fiducial markers.

**Figure 2:**
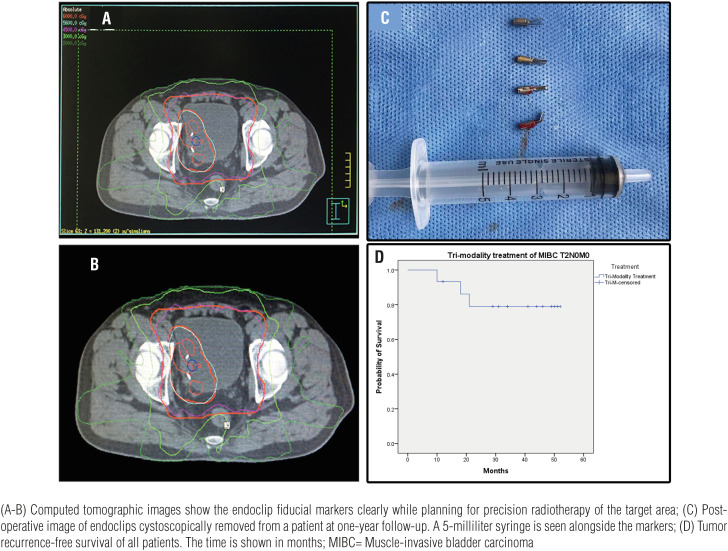
Precision radiotherapy planning and follow-up, Kaplan-Meier Curve. (A-B) Computed tomographic images show the endoclip fiducial markers clearly while planning for precision radiotherapy of the target area; (C) Post-operative image of endoclips cystoscopically removed from a patient at one-year follow-up. A 5-milliliter syringe is seen alongside the markers; (D) Tumor recurrence-free survival of all patients. The time is shown in months; MIBC= Muscle-invasive bladder carcinoma

The procedure was performed in a fully equipped urology theater using a Cystoscope NP-3 (22.5Fr, 3.5mm operating port, Shenyang University Endoscope Co. Ltd, China). The bladder was carefully inspected for the location of the tumor relative to the adjacent structures before resection. Comprehensive tumor excision was carried out to the deep muscular layer of the bladder wall, removing the base of the tumor completely. As fiducial markers for image-guided precision radiotherapy, two to five titanium endoclips (Olympus HX-600-135, Olympus Inc., Japan) (Figure-1A) were applied through the operating port to the excised edge of the bladder muscular wall (Figures 1B and C). X-ray imaging of the kidney-ureter-bladder was done postoperatively to confirm the presence of the endoclips (Figure-1D). A three-way Foley catheter (18Fr) was inserted for irrigation and was removed a week after the transurethral resection of the bladder tumor. All patients underwent one round of radio-sensitizing chemotherapy 3 weeks after transurethral resection of the bladder tumor. The same chemotherapy regimen (Gemcitabine 1g/m^2^, Cisplatin 70mg/m^2^) was used in all patients. Image-guided radiotherapy and whole-bladder radiotherapy were initiated one month after resection of the bladder tumor. The image-guided radiotherapy protocol (5600∼5800 centigray) was given to the tumor location by computed tomographic planning (Figures 2A and B), and to the whole bladder (4500∼5000 centigray) along with systemic chemotherapy. The chemotherapy (intravesical infusion of chemotherapy drugs, berubicin and gemcitabine) was initiated within 24 hours after surgery, then given once a week for 8 consecutive weeks, then once a month for 1 year.

### Follow-up

A follow-up electronic cystoscopy was performed once every 3 months for a total of 4 times during the 1^st^ year after the procedure. At one-year post resection and adjuvant chemoradiotherapy, the endoclips were removed on follow-up cystoscopy (Figure-2C). Follow-up computed tomography and/or color doppler ultrasound were done every 3 months after discharge for 9 months, and then annually. After the first year, patients were followed at their local hospitals. The patient's status was inquired on phone after one year, as they did not follow-up at our clinic, and all post-annual evaluations were made at their local hospitals. The telephonic inquiry included questions about urination, hematuria, dysuria, urgency, frequency, and change in the urine stream and any abnormal findings on imaging studies. Patients who had a recurrence during follow-up were treated with salvage radical cystectomy. Any procedure-related complications, loss or migration of the fiducial markers, recurrence of the tumor, and cause of death were recorded and analyzed. Procedure-related complications were defined as postoperative hematuria, cystitis, urinary tract infection, obvious ischemic necrosis of bladder mucosal tissue, obvious symptoms of bladder irritation after catheter removal, chronic pain, dysuria, loss of titanium clip markers, inability to remove titanium clip markers. Precision radiation-related complications were regarded as radiation cystitis and proctitis. Chemotherapy related complications were considered as effects on the hematologic system, hematopoietic system, nausea, and vomiting.

### Statistical analysis

The data are presented as mean±standard deviation and range (in parentheses). The recurrence-free survival is presented as a Kaplan-Meier curve (IBM SPSS statistics 21).

## RESULTS

Fifteen patients diagnosed with T_2_N_0_M_0_ stage muscle-invasive bladder carcinoma were enrolled in the study. Patient characteristics are given in Table-1. All 15 patients underwent successful, visibly complete resection of the tumor. The histopathology reports showed that 13/15 patients had infiltrative high-grade urothelial carcinoma, while 1/15 had high grade intraepithelial urothelial carcinoma, and 1/15 had poorly differentiated carcinoma tending towards high grade infiltrative urothelial with squamous and sarcomatoid differentiation.

In total, 49 fiducial markers (endoclips) were placed in all patients (range 2-5) to facilitate image-guided radiotherapy post-resection. All 49 endoclips were visible post-resection and at the last radiotherapy treatment. All 49 endoclips were removed after one year of follow-up. Importantly, none of the endoclips were lost or shifted after placement.

The total hospitalization cost was 18555.4±4581.1 Chinese yuan (range 9565.8-26073) (*). The endoclip costs 186 Chinese yuan per clip. The mean number of follow-up months was 38.9±13.2 (range 11-52). There were no procedure-related complications at discharge or follow-up. There we no reports of post-discharge hematuria, dysuria, cystitis, and pain. There were no reported major complications of radiotherapy. The common complaint post-chemotherapy was nausea and vomiting. At one-year after completion of therapy, the overall recurrence free survival rate was 93.3% and overall survival rate was 100% (Figure-2D). Two patients had a recurrence at 10 and 18 months, respectively, post-surgery; both underwent salvage radical cystectomy and no further recurrence was noted at the latest follow-up. Another patient died of cancer cachexia 9 months after completion of therapy. Computed Tomographic examination showed retroperitoneal metastasis (highly likely to be of bladder origin), but no new tumor in the bladder was seen. No autopsy was performed and the source of the tumor could not be determined.

## DISCUSSION

Muscle-invasive bladder carcinoma has a high recurrence and mortality rate. Radical resection of the bladder tumor and chemoradiotherapy, termed trimodality bladder-preserving therapy, are the main methods of bladder preservation. Radical cystectomy has been the standard of care for muscle-invasive bladder carcinoma for a long time. A study of modern radical cystectomy (with pelvic radiotherapy) reported a survival rate of ∼66% (14). To date, there are no completed randomized studies comparing radical cystectomy and bladder-preserving trimodality treatment, but there are multiple studies that favor bladder-preserving trimodality treatment in well-selected patients (7, 15). The availability of different choices with similar outcomes can give a surgeon ease of mind in decision making. Our results promise another option in the bladder preserving procedure.

The total cost of surgery is lower, which adds to the favorable outcome of the procedure. The application of the endoclips was easy and no complications were reported due to the endoclips, which adds to the safety of the procedure. We removed all the endoclips after completion of the therapy and this further elaborates the device usability, as a potential source of inflammation is removed without any consequences.

### Endoclips versus current fiducial markers

The current fiducial markers Lipiodol and gold seeds are being used successfully for target registration, and with very few reported complications. Here we summarize a comparison of endoclips versus Lipiodol and gold seeds.

Endoclips: The endoclips we successfully used as fiducial markers showed very good biocompatibility in the bladder. The application method for this novel fiducial markers is very safe and simple. Both insertion and removal of the endoclips are easily accomplished by cystoscopy. In our experience, we noticed that all the markers were firmly in place, even at removal, thus, they are not likely to dislodge spontaneously. Moreover, they were easy to apply at various anatomical sites all over the bladder, and there were no post-application complications. As with the currently used fiducial markers, the novel endoclip markers provided accurate target positioning. The application of the endoclips was achieved by using a rigid cystoscope (Figure-2). We have not yet been able to apply these markers by using a flexible cystoscope. To our knowledge, there are no contraindications for endoclip application after transurethral resection of bladder tumor.

Lipiodol: The Lipiodol fiducial marker is applied with a flexible cystoscope. Similar to endoclips, Lipiodol marks do not move. However, the different sizes and shapes of the Lipiodol marks make it difficult to control target radiotherapy, and this can result in bladder mucosal damage and deformation. Moreover, Lipiodol is difficult to inject near the neck of the bladder, in contrast to endoclips, which can be applied to any bladder site. Finally, Lipiodol is contraindicated in hyperthyroidism.

Gold seeds: These also facilitate accurate positioning, and no adverse reactions have been reported. However, placement at the bladder dome and neck can be difficult (12) and the seeds can become dislodged. They may also be invisible on computed tomography.

From this summary, endoclips have clear advantages as a novel fiducial marker. Our results are from well-selected patients who underwent transurethral resection of bladder tumor followed by chemoradiotherapy. With the help of endoclips for guided radiotherapy, 13 of 15 (86.6%) of our patients achieved a complete remission at one year. In our study, all patients were in clinical stage T2, which likely contributed to our optimal results, as patients with clinical stage T2 are known to have higher remission rates as compared to other clinical stages (T3, T4) (16).

### Study limitations

Our patient sample size is small, and we did not have a control group. One of our patients died from metastasis. Larger studies of patients with a range of clinical stages are needed to compare endoclips with currently used fiducial markers for their help in oncologic outcomes.

## CONCLUSIONS

The use of endoclips as a novel fiducial marker for precision radiotherapy following tumor resection in patients with clinical stage T2 muscle-invasive bladder carcinoma is feasible. Our patient cohort showed favorable outcomes with the use of endoclips. Endoclips are easy to apply, effective as markers, inexpensive and apparently safe. We recommend their use as fiducial markers to enhance the outcomes of precision radiotherapy.

## Footnotes

(*) Reviewer's note: 1 US dollar=approximately 7 Chinese yuan

## References

[1] 1. Antoni S, Ferlay J, Soerjomataram I, Znaor A, Jemal A, Bray F. Bladder Cancer Incidence and Mortality: A Global Overview and Recent Trends. Eur Urol. 2017;71:96-108. 10.1016/j.eururo.2016.06.010 27370177

[2] 2. Ferlay J, Soerjomataram I, Ervik M, Dikshit R, Eser S, Mathers C, et al. GLOBOCAN 2012. Estimated cancer incidence, mortality and prevalence worldwide in 2012. 10.1002/ijc.29210 25220842

[3] 3. Bladder cancer incidence statistics. Available from: http://www.cancerresearchuk.org/cancer-info/cancerstats/types/bladder/incidence/.

[4] 4. Greiman AK, Rosoff JS, Prasad SM. Association of Human Development Index with global bladder, kidney, prostate and testis cancer incidence and mortality. BJU Int. 2017;120:799-807. 10.1111/bju.13875 28480994

[5] 5. Chang SS, Bochner BH, Chou R, Dreicer R, Kamat AM, Lerner SP, et al. Treatment of Nonmetastatic Muscle-Invasive Bladder Cancer: American Urological Association/American Society of Clinical Oncology/American Society for Radiation Oncology/Society of Urologic Oncology Clinical Practice Guideline Summary. J Oncol Pract. 2017;13:621-625. 10.1200/JOP.2017.024919 28796558

[6] 6. Gofrit ON, Nof R, Meirovitz A, Pode D, Frank S, Katz R, et al. Radical cystectomy vs. chemoradiation in T2-4aN0M0 bladder cancer: a case-control study. Urol Oncol. 2015;33:19.e1-19.e5. 10.1016/j.urolonc.2014.09.014 25445384

[7] 7. Efstathiou JA, Spiegel DY, Shipley WU, Heney NM, Kaufman DS, et al. Long-term outcomes of selective bladder preservation by combined-modality therapy for invasive bladder cancer: the MGH experience. Eur Urol. 2012;61:705-11. 10.1016/j.eururo.2011.11.010 22101114

[8] 8. Lotz HT, Pos FJ, Hulshof MC, van Herk M, Lebesque JV, Duppen JC, et al. Tumor motion and deformation during external radiotherapy of bladder cancer. Int J Radiat Oncol Biol Phys. 2006;64:1551-8. 10.1016/j.ijrobp.2005.12.025 16580504

[9] 9. Muren LP, Smaaland R, Dahl O. Conformal radiotherapy of urinary bladder cancer. Radiother Oncol. 2004;73:387-98. 10.1016/j.radonc.2004.08.009 15588887

[10] 10. Cowan RA, McBain CA, Ryder WD, Wylie JP, Logue JP, Turner SL, et al. Radiotherapy for muscle-invasive carcinoma of the bladder: results of a randomized trial comparing conventional whole bladder with dose-escalated partial bladder radiotherapy. Int J Radiat Oncol Biol Phys. 2004;59:197-207. 10.1016/j.ijrobp.2003.10.018 15093917

[11] 11. Baumgarten AS, Emtage JB, Wilder RB, Biagioli MC, Gupta S, Spiess PE. Intravesical lipiodol injection technique for image-guided radiation therapy for bladder cancer. Urology. 2014;83:946-50. 10.1016/j.urology.2013.09.058 24397940

[12] 12. Mangar S, Thompson A, Miles E, Huddart R, Horwich A, Khoo V. A feasibility study of using gold seeds as fiducial markers for bladder localization during radical radiotherapy. Br J Radiol. 2007;80:279-83. 10.1259/bjr/54321311 17121759

[13] 13. Meijer GJ, van der Toorn PP, Bal M, Schuring D, Weterings J, de Wildt M. High precision bladder cancer irradiation by integrating a library planning procedure of 6 prospectively generated SIB IMRT plans with image guidance using lipiodol markers. Radiother Oncol. 2012;105:174-9. 10.1016/j.radonc.2012.08.011 23022177

[14] 14. Zehnder P, Studer UE, Skinner EC, Dorin RP, Cai J, Roth B, et al. Super extended versus extended pelvic lymph node dissection in patients undergoing radical cystectomy for bladder cancer: a comparative study. J Urol. 2011;186:1261-8. 10.1016/j.juro.2011.06.004 21849183

[15] 15. Hoskin PJ, Rojas AM, Bentzen SM, Saunders MI. Radiotherapy with concurrent carbogen and nicotinamide in bladder carcinoma. J Clin Oncol. 2010;28:4912-8. 10.1200/JCO.2010.28.4950 20956620

[16] 16. Giacalone NJ, Niemierko A, Shipley WU, Efstathiou JA. Reply to Saeid Safiri and Erfan Ayubi's Letter to the Editor re: Nicholas J. Giacalone, William U. Shipley, Rebecca H. Clayman, et al. Long-term Outcomes After Bladder-preserving Tri-modality Therapy for Patients with Muscle-invasive Bladder Cancer: An Updated Analysis of the Massachusetts General Hospital Experience. Eur Urol 2017;71:952-60. Methodological Issues to Avoid Misinterpretation. Eur Urol. 2017;72:e64-e65. 10.1016/j.eururo.2017.06.007 28642020

